# A 14‐Year Study of Serum Glial Fibrillary Acidic Protein and Total Tau in Premanifest Huntington's

**DOI:** 10.1002/acn3.70057

**Published:** 2025-04-21

**Authors:** Natalia E. Owen, Zanna J. Voysey, Jonathan A. Holbrook, Maura Malpetti, Clara Le Draoulec, Lennart R. B. Spindler, Anna O. G. Goodman, Alpar S. Lazar, Roger A. Barker

**Affiliations:** ^1^ Department of Clinical Neurosciences, John van Geest Centre for Brain Repair University of Cambridge Cambridge UK; ^2^ Department of Clinical Neurosciences, Cambridge Centre for Frontotemporal Dementia and Related Disorders University of Cambridge Cambridge UK; ^3^ UK Dementia Research Institute at University of Cambridge Cambridge UK; ^4^ Faculty of Medicine and Health Sciences University of East Anglia Norwich UK; ^5^ Cambridge Stem Cell Institute, University of Cambridge Cambridge UK

**Keywords:** blood‐based biomarkers, glial fibrillary acidic protein, Huntington's disease, prognostic biomarkers, tau

## Abstract

There is a pressing need for blood biomarkers that can identify Huntington's disease (HD) gene carriers' proximity to manifest disease. We previously examined serial serum neurofilament light (NfL) concentrations in 21 premanifest HD gene carriers and 14 controls over 14 years, finding that NfL demonstrates high prognostic value and distinct longitudinal dynamics in premanifest/transitional HD. Here, we report the corresponding results for serum glial fibrillary acidic protein and total tau, providing the first longitudinal and head‐to‐head study of these biomarkers in HD. Our findings do not support the utility of these analytes as prognostic biomarkers in premanifest and transitional HD.

## 1 Introduction

Huntington's disease (HD) is a fully penetrant, monogenic neurodegenerative condition, manifesting with psychiatric, cognitive and motor features, for which we have no disease‐modifying treatments. Age of onset is highly variable, which cannot be entirely accounted for by existing measures. Biomarkers capable of predicting proximity to disease onset are therefore a pressing need, both to improve prognostication for patients and to enhance participant selection for clinical trials of emerging disease‐modifying therapies.

Recent research has sought to address this by evaluating the efficacy of fluid biomarkers in HD. The most prominent of these include (i) neurofilament light (NfL), a cytoskeletal protein released following axonal damage or neuronal death, (ii) total tau (t‐tau), a microtubule‐associated protein released following neuronal death and (iii) glial fibrillary acidic protein (GFAP), an intermediate filament protein elevated in the setting of reactive astrocytosis. All three are known to become elevated in both blood and cerebrospinal fluid (CSF) in several neurodegenerative diseases [1, 2, 3, 4], including manifest HD [5, 6, 7, 8, 9], and have shown some prognostic potential prior to overt disease manifestation in Alzheimer's disease [4, 10]. NfL has been found to become elevated in premanifest HD gene carriers, and 3‐year longitudinal studies have suggested its potential to predict the risk of conversion to manifest HD. CSF GFAP has also been shown to correlate with a 5‐year risk of disease onset in premanifest HD gene carriers [6]. By contrast, t‐tau has not yet exhibited prognostic potential in premanifest HD, but further investigation is supported by the fact that CSF tau levels correlate with clinical features in manifest HD [7], tau pathology is over‐represented in post‐mortem HD brains [11, 12, 13], and tau immunisation has been found to improve clinical outcomes in HD mouse models [14].

One of the key ‘bottlenecks’ in establishing the true prognostic utility of these analytes has been a lack of head‐to‐head comparison studies, and a lack of extended serial longitudinal studies. We recently sought to address this by reporting the longitudinal dynamics and predictive power of serum NfL in premanifest and transitional HD patients, through a 14‐year study over four timepoints [15]. We showed that, in HD gene carriers within approximately 10 years of disease onset, serum NfL levels are significantly elevated compared to both controls and gene carriers > 10 years from disease onset, and then increase more rapidly across time thereafter. As such, a baseline timepoint serum NfL cut‐off of 24.06 pg/mL was found to identify those who would phenoconvert during the study period with 100% sensitivity and specificity. Alongside this, a higher rate of change in NfL was found to be predictive of more severe motor and cognitive impairments at study completion. Here, we expand our previous results by investigating the corresponding trajectories and prognostic value of serum GFAP and t‐tau from the same cohort of individuals. This constitutes the first longitudinal study of GFAP and t‐tau dynamics in HD, and the first study to analyse these three fluid biomarkers concomitantly in HD.

## 2 Methods

The methods of the study are previously described in Voysey et al. [15]. In brief, 21 HD gene carriers and 14 controls underwent blood sampling at four timepoints across a 14‐year period (Baseline: 2009–2010, Time 2: 2011–2012, Time 3: 2013–2014, Time 4: 2022–2023). All HD gene carriers were premanifest at baseline. Participants underwent assessment for motor features of HD at each timepoint as well as cognitive assessment at baseline and Time 4. Motor assessment comprised the Unified Huntington's Disease Rating Scale (UHDRS); cognitive assessment comprised Montreal Cognitive Assessment (MoCA), Trails test A and B, Symbol Digit Modalities Test (SDMT) and Semantic and Phonemic fluency tasks. Participants had no history of traumatic brain injury, renal impairment, neuroinflammatory or neurodegenerative disease (apart from HD in gene carriers) throughout the study period and were age < 65 at baseline. Serum GFAP and t‐tau concentrations were obtained using the same methodology as was used for NfL: namely, the Meso Scale Discovery S‐PLEX Neurology Panel 1 (Human) [15]. All statistical analyses were adjusted for age, sex, CAG repeat length and body mass index (BMI), and multiple comparisons in regressions were controlled for via Benjamini‐Hochberg correction.

## 3 Results

The demographic profiles and clinical trajectories of participants are provided in full in Voysey et al. [15]. All 21 HD gene carriers remained premanifest at Time 2, but 2/21 had become prodromal/manifest by Time 3. Baseline motor and cognitive profiles did not differ between groups [15]. By Time 4, 14/21 had converted to prodromal/manifest HD (‘converters’), whereas 7/21 gene carriers remained premanifest (‘non‐converters’). No participant progressed to moderate/advanced HD.

Some participants elected to omit a proportion of cognitive tests or some blood sampling timepoints. Considering all participants and timepoints, GFAP and t‐tau data was available in 69% of possible observations, and clinical scores were available in 79%. Missingness was distributed equally among groups, apart from comparatively less GFAP and t‐tau data among non‐converters at baseline (*p* = 0.025). Participants withdrawing at Time 4 exhibited lower predicted years to onset at baseline (*p* < 0.001) [15]. Despite this, they did not differ from non‐withdrawals with respect to baseline or annualised rate of change in GFAP or t‐tau levels, arguing against the influence of selective attrition. We further mitigated against the potential effects of missing data by incorporating bootstrapping and linear mixed models.

**FIGURE 1:**
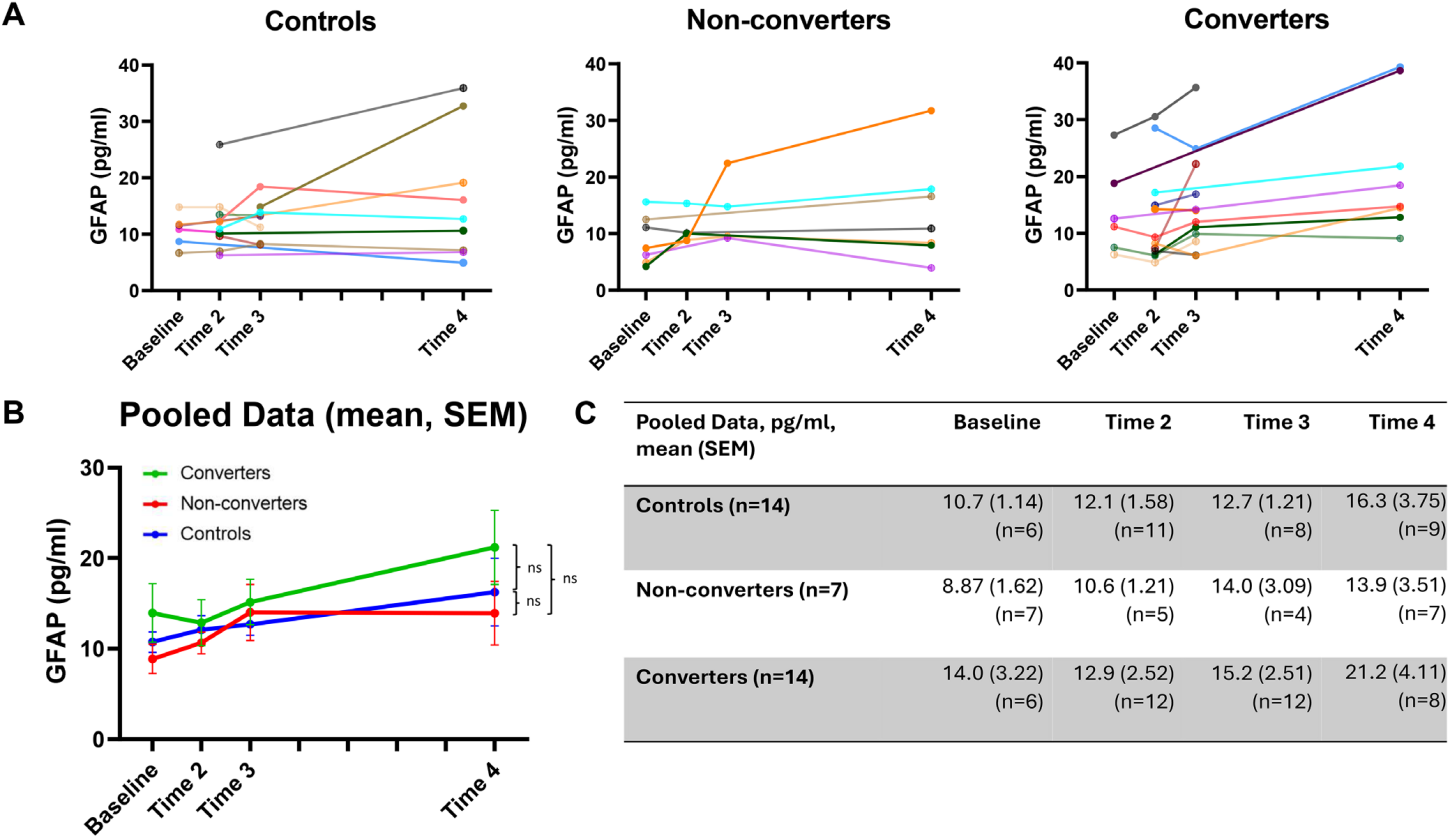
Longitudinal dynamics of GFAP concentrations by group. (A) Longitudinal dynamics of GFAP concentrations for individual participants. Left shaded markers indicate sampling in the first half of a given timepoint period, and right shaded markers indicate sampling in second half of a given timepoint period. (B) Overall longitudinal dynamics of GFAP concentrations for each participant group. Error bars = SEM. Ns = *p* > 0.05 in group*time interaction adjusted for age, sex, CAG repeat length and BMI. (C) Table displaying means and SEM of GFAP concentrations for each participant group.

**FIGURE 2:**
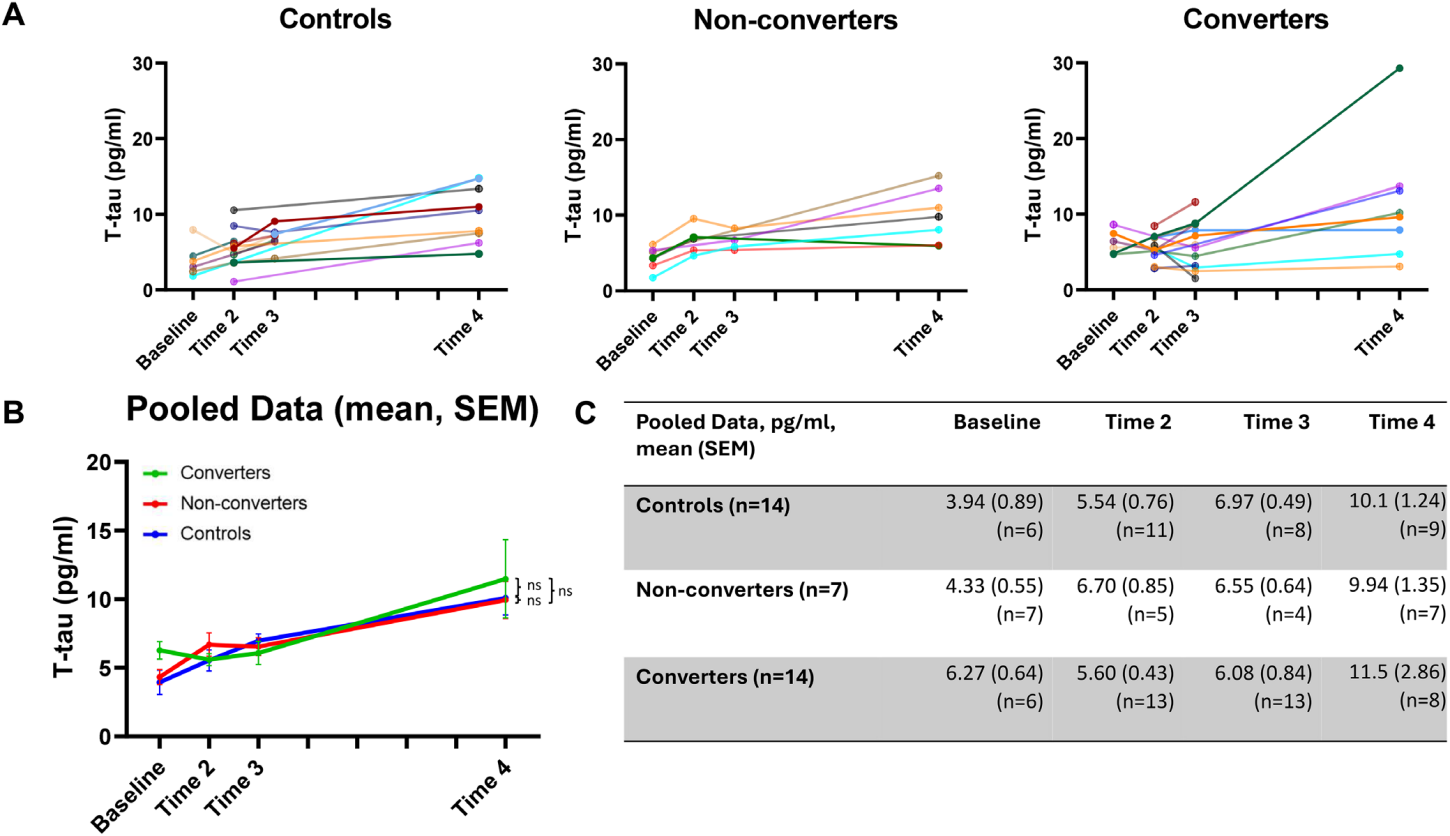
Longitudinal dynamics of t‐tau concentrations by group. (A) Longitudinal dynamics of t‐tau concentrations for individual participants. Left shaded markers indicate sampling in the first half of a given timepoint period, and right shaded markers indicate sampling in second half of a given timepoint period. (B) Overall longitudinal dynamics of t‐tau concentrations for each participant group. Error bars = SEM. Ns = *p* > 0.05 in group*time interaction adjusted for age, sex, CAG repeat length and BMI. (C) Table displaying means and SEM of t‐tau concentrations for each participant group.

In cross‐sectional analysis of group differences via ANCOVA at each timepoint, mean serum concentrations of GFAP were higher in converters than non‐converters and controls at each timepoint, but this did not reach statistical significance in any instance (Baseline: *F* = 1.46, *p* = 0.26, *ƞ* ^2^ = 0.15; Time 2: *F* = 0.16, *p* = 0.85, *ƞ*_p_^2^ = 0.013; Time 3: *F* = 0.028, *p* = 0.97, *ƞ*_p_^2^ = 0.003; Time 4: *F* = 0.35, *p* = 0.71, *ƞ*_p_^2^ = 0.05) (Figure 1A–C). Similarly, t‐tau levels did not differ significantly between groups at any timepoint (Baseline: *F* = 3.12, *p* = 0.072, *ƞ* ^2^ = 0.28; Time 2: *F* = 2.09, *p* = 0.15, *ƞ*_p_^2^ = 0.14; Time 3: *F* = 0.34, *p* = 0.72, *ƞ* ^2^ = 0.030; Time 4: *F* = 0.41, *p* = 0.67, *ƞ*_p_^2^ = 0.003; Figure 2A–C). Upon repeating analysis incorporating bootstrapping (5000 replicates) all results remained non‐significant.

Likewise, longitudinal analysis did not suggest any difference in the serial dynamics of serum GFAP or t‐tau concentrations across the study period between controls, non‐converters and converters. Linear mixed effects modelling indicated no significant group or group*time interaction for either analyte (Figures 1B and 2B). The estimated annualised rate of change for both analytes also did not differ significantly between groups (GFAP, converters 0.62 (±0.44) pg/mL/year, non‐converters 0.39 (±0.68) pg/mL/year, controls 0.23 (±0.36) pg/mL/year; t‐tau, converters 0.46 (±0.64) pg/mL/year, non‐converters 0.44 (±0.23) pg/mL/year, controls 0.39 (±0.25) pg/mL/year) with *p* values ranging from 0.27 to 0.99.

**FIGURE 3:**
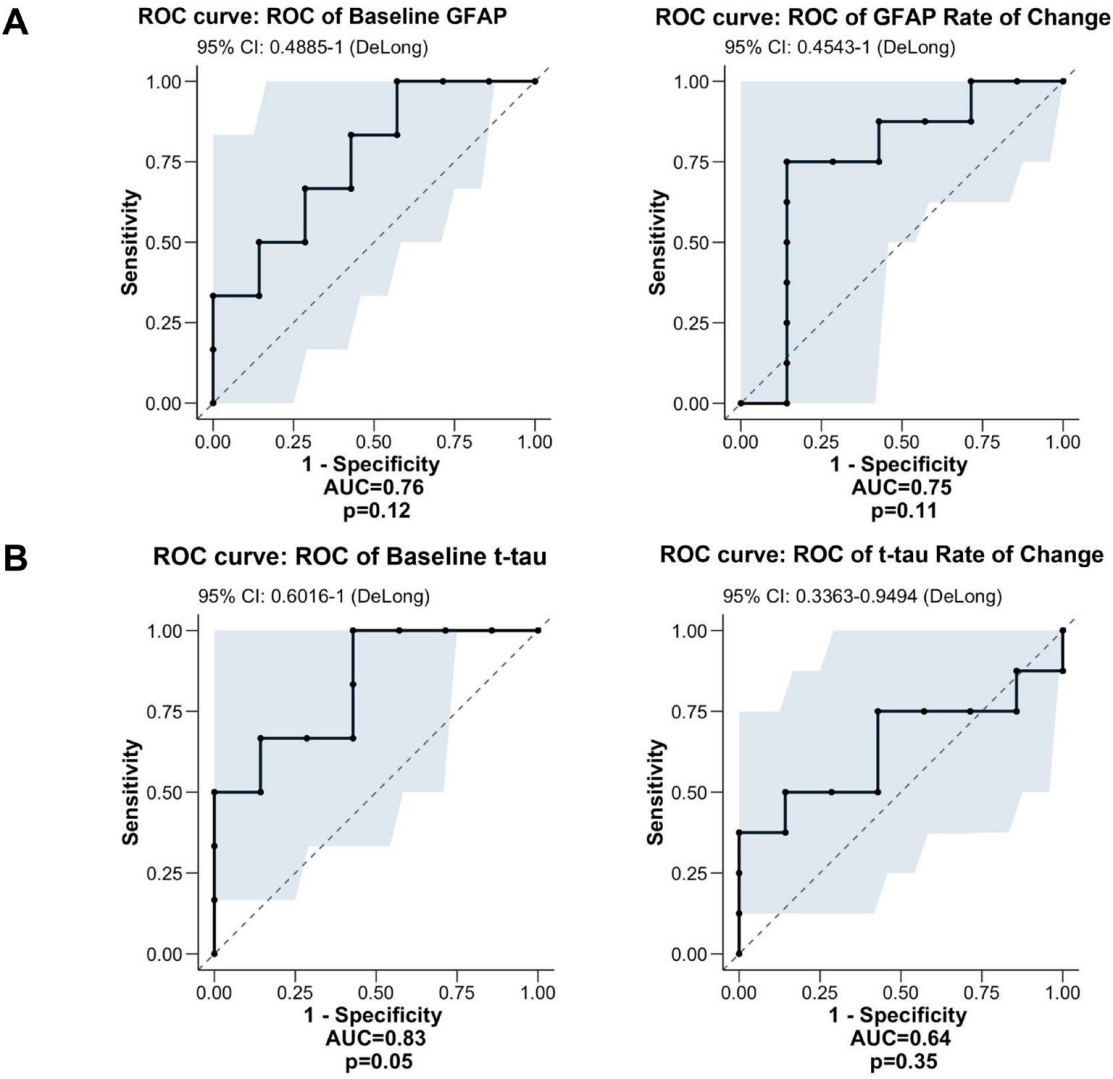
ROC curve analyses of baseline and annualised rate of change in (A) GFAP concentrations and (B) t‐tau concentrations versus discrimination of converter from non‐converter gene carriers. AUC, area under curve.

In regression analyses, baseline GFAP concentrations were predictive of more severe UHDRS total motor scores among gene carriers at study completion (*R* ^2^ = 0.66, *β* = 1.55, *p* = 0.0008). Otherwise, however, there were no significant associations between either GFAP or t‐tau levels for any motor or cognitive assessment at any timepoint. Likewise, the annualised rate of change in GFAP or t‐tau levels in gene carriers was not predictive of any motor or cognitive outcome at study completion. Moreover, for both GFAP and t‐tau, receiver operating characteristic (ROC) curve analysis indicated that neither baseline measurements nor annualised rate of change exhibited significant efficacy in discriminating between gene carriers who converted to prodromal/manifest disease during the study and those who remained premanifest (Figure 3A,B). Although AUC values were relatively high, this did not reach statistical significance in any case.

## 4 Discussion

Both cross‐sectionally and longitudinally, our results do not support strong prognostic or disease‐tracking abilities of either serum GFAP or t‐tau in premanifest and transitional HD. This contrasts with our parallel findings regarding NfL, which suggested significant prognostic potential in this disease stage. The only exception to this was in relation to higher baseline serum GFAP predicting more severe motor features at study completion.

Such results corroborate several recent studies that suggest serum GFAP and t‐tau only show elevations in more moderate to advanced stages of HD and, thus, are better for tracking disease severity and progression after motor onset, particularly in comparison with NfL [5, 9, 16, 17]. Our study population did not include these stages of HD, which likely accounts for our findings.

The biological mechanisms driving these temporal variations in biofluid markers remain unexplained but are consistent with predominantly neuronal damage occurring in premanifest/early stages of HD [18], versus progressive glial dysfunction and abnormal tau accumulation in later stages. This hypothesis is supported by findings of post‐mortem studies, where tau pathology and astrogliosis have been predominantly restricted to later stages of HD [12, 19, 20]. As demonstrated in a preclinical HD model, increased GFAP expression may be indicative of reactive astrocytosis facilitating the clearance of mutant huntingtin aggregates [21].

Our results suggest that serum GFAP may be slightly superior to t‐tau as a biomarker for disease activity in HD. This is consistent with the aforementioned studies [6] and cross‐sectional studies reporting associations between plasma GFAP and more severe motor scores among other clinical measures of disease severity [5, 16], whereas plasma t‐tau exhibits weaker, or no, associations [22, 23]. This may in part be because t‐tau predominantly reflects peripheral sources of tau. Future studies using newer brain‐derived tau assays, which have been shown to correlate strongly with CSF tau levels, may therefore generate alternative findings [24].

Given the low power and proportion of missing data within the study, these results should be interpreted as exploratory and a precursor to larger, more robust validation studies. Nevertheless, this study extends the literature by helping to elucidate (i) the value of fluid biomarkers in HD prognostication and (ii) the underlying pathobiology in premanifest and transitional HD. Overall, our results do not support the use of serum GFAP or t‐tau as prognostic biomarkers in premanifest and transitional HD and specifically positions them as inferior to NfL in this regard. Instead, their utility is likely to be restricted to more advanced stages of HD.

## Author Contributions

Conceptualization: Z.J.V., R.A.B. Sample collection: A.O.G.G., A.S.L., Z.J.V., N.E.O. Sample analysis: J.A.H. Data analysis: Z.J.V., N.E.O., M.M., L.R.B.S., C.L.D. Manuscript drafting: N.E.O. Figures: N.E.O. Manuscript revision: R.A.B., A.S.L., Z.J.V., N.E.O., A.O.G.G., J.A.H., L.R.B.S., M.M., C.L.D. Supervision: R.A.B.

## Conflicts of Interest

The authors declare no conflicts of interest.

## Data Availability

The data that support the findings of this study are available upon request from the corresponding author. The data are not publicly available due to privacy or ethical restrictions.
